# Visual and Anatomical Outcomes of a Single Intravitreal Dexamethasone in Diabetic Macular Edema: An 8 Year Real-World Study

**DOI:** 10.3390/jcm12123878

**Published:** 2023-06-06

**Authors:** Livia Faes, Amit V. Mishra, Veronika Lipkova, Konstantinos Balaskas, Chrystie Quek, Robin Hamilton, Ulrike Held, Dawn Sim, Sobha Sivaprasad, Dun Jack Fu

**Affiliations:** 1 NIHR Biomedical Research Centre, Moorfields Eye Hospital NHS Foundation Trust, UCL Institute of Ophthalmology, London EC1V 9EL, UK; l.faes@nhs.net (L.F.); k.balaskas@nhs.net (K.B.); chrystiequek@gmail.com (C.Q.); robin.hamilton1@nhs.net (R.H.); dawnasim@nhs.net (D.S.); sobha.sivaprasad@nhs.net (S.S.); 2 Kings College London, London WC2R 2LS, UK; vero.lipkova@gmail.com; 3 Epidemiology, Biostatistics and Prevention Institute, University of Zurich, 8057 Zurich, Switzerland; ulrike.held@uzh.ch; 4 Genentech Roche, 1 DNA Way, South San Francisco, CA 940980, USA

**Keywords:** visual outcome, survival analysis, diabetic macular edema

## Abstract

Importance: Diabetic macular edema (DME) is a major cause of vision loss in patients with diabetes mellitus. Intravitreal dexamethasone is a treatment option for patients unsuitable for or non-responsive to anti-angiogenic agents. Objective: To quantify visual and anatomical outcomes from an initial intravitreal dexamethasone injection over the expected 6-month period of dexamethasone release by the implant. Design and enrolment: This is a retrospective cohort study using electronic medical records of patients reviewed between 1 January 2012 and 1 April 2022. Setting: A tertiary eye-care center in London, United Kingdom; Moorfields Eye Hospital National Healthcare System Foundation Trust. Participants: The cohort comprised 418 adult patients with DME who received an initial treatment of 700 µg intravitreal dexamethasone in the study period. Of these, 240 patients met the inclusion criteria of ≥2 hospital visits following initial injection (≥1 beyond 6 months) and no previous ocular corticosteroid treatment or missing assessment at baseline. Exposure(s): Intravitreal dexamethasone implant (700 µg). Main Outcome(s) and Measure(s): Probability of a positive visual outcome, defined as ≥5 or ≥10 Early Treatment Diabetic Retinopathy Study (ETDRS)-letter gain after treatment when compared to baseline (Kaplan–Meier models). Results: From the initial intravitreal dexamethasone injection alone, we observed a >75% chance of gaining ≥5 ETDRS letters and >50% chance of gaining ≥10 ETDRS letters within 6 months. There was less than a 50% chance of sustaining either positive visual outcome beyond 4 months. Conclusions and Relevance: Most patients can be expected to have a positive visual outcome following an initial injection of dexamethasone implants that subsides within 4 months. Real-world re-treatment was observed to be delayed until after visual benefits were lost in half of the cohort. Further research will be needed to study the effects of delays in re-treatment.

## 1. Introduction

The number of people with diabetes mellitus is estimated to reach over 500 million within the next ten years [1]. Vascular complications frequently associated with progressive disease can manifest in the eye and have deleterious effects on vision. Diabetic eye disease is the most common cause of visual disability in the working-age population worldwide [2,3,4]. There are currently 21 million patients with diabetic eye disease worldwide, which is expected to increase with the projected prevalence of diabetes from 415 million in 2015 to 642 million in 2040 [5].

Diabetic macular oedema (DME) is the most common form of sight-threatening diabetic eye disease, in which fluid leaking from damaged retinal blood vessels accumulates in the macula, the central part of the retina, which is responsible for high-resolution visual acuity and color vision. Chronic edema of the macula can irreversibly damage both inner and outer retina layers. The overall risk of DME in patients with diabetes is currently estimated at 7% (and at 29% after 20 years of disease duration), thus establishing it as a major cause of vision loss in diabetic patients [6].

First-line treatment for DME is intravitreal anti-vascular endothelial growth factor (VEGF). Randomized controlled trials (RCTs) have demonstrated that intravitreal injections with anti-VEGF agents improve the prognosis of patients with DME in terms of visual acuity (VA) when following a fixed-interval treatment regimen. However, up to 40% of patients show incomplete visual or anatomical response to anti-angiogenic agents [7]. Intravitreal steroid implants are anti-inflammatory approaches to treating DME in patients who are pseudophakic or who are considered insufficiently responsive to or unsuitable for anti-angiogenic therapy. Intravitreal steroids currently include dexamethasone or fluocinolone implants.

Intravitreal dexamethasone implants were approved for use in adult patients with DME in 2014 [8,9,10]. The biodegradable solid-polymer drug-delivery system releases dexamethasone over a period of 6 months [11]. The product label in Europe recommends re-treatment after approximately 6 months in patients with an initial response when the patient experiences decreased vision and/or an increase in retinal thickness, secondary to recurrent or worsening diabetic macular edema. The Diabetic Retinopathy Clinical Research Network (DRCR.net)’s Protocol U suggested that patients with persistent vision loss and edema following 6 months to 1 year of monthly anti-VEGF treatment may benefit from adjunctive corticosteroid therapy [12].

RCTs evaluating intravitreal dexamethasone in DME primarily report visual outcomes by averaging visual acuity (VA) and retinal thickness at predefined time points—for example, mean VA at month 4 [9,12]—which is largely mirrored by retrospective studies of clinical-practice data, i.e., real-world studies [13,14,15]. In comparison to real-world studies, RCTs typically feature limited missing data (9% of RCTs between July and December 2013) [16], and with appropriate methodology and execution, missing observations can be assumed to occur randomly. Thus multiple imputation models for missing data and mixed-effects models for outcome estimates are generally accepted [17]. Yet these techniques are rarely applicable to real-world studies. Here, the circumstances of missing observations are seldom described, and the underlying reasons cannot be assumed to be random; thus, generalizing available data to the target population is prone to survival bias. It has been proposed that time-to-event analyses, such as Kaplan–Meier survival and Cox proportional-hazards regression tests, address some of these limitations by making use of all available data through the extrapolation of outcome probabilities [18]. Furthermore, these techniques promote the consideration of clinically relevant outcomes, which means that VA at an arbitrary time point is inherently not. Thus far, real-world studies of intravitreal dexamethasone in DME have yet to evaluate visual outcomes using time-to-event analyses.

The objective of the present study was to use time–event methodologies to quantify real-world, clinically significant visual outcomes from an initial intravitreal dexamethasone injection over the expected period of 6 months of dexamethasone release by the implant in a tertiary eye-care center in the United Kingdom (UK).

## 2. Methods

### 2.1. Study Design and Setting

This retrospective cohort study was conducted at Moorfields Eye Hospital National Health Service (NHS) Foundation Trust, a tertiary center in London, UK. The study was conducted in compliance with the Declaration of Helsinki, the Institutional Review Board of Moorfields Eye Hospital—the Moorfields Eye Hospital Clinical Audit Department (CA21/MR/1026), and was reported in accordance with the Strengthening the Reporting of Observational Studies in Epidemiology (STROBE) reporting guideline. Informed consent from the study cohort was not required as per the standard when using retrospective, deidentified data for research within the UK NHS.

### 2.2. Cohort

The cohort comprised patients with DME that received an initial intravitreal injection of 700 µg dexamethasone (Ozurdex^®^: Allergan, Inc., Irvine, CA, USA) between 1 January 2012 and 1 April 2022. Patients ≥18 years of age were required to have at least two ophthalmic visits following the initial injection, with at least one falling beyond 6 months. Patients with previous intraocular or periocular steroid treatment or missing visual acuity and optical-coherence tomography at baseline were excluded from the study. If both eyes were injected, one was selected at random using the sample function of base R software, version 3.6.2 (R Foundation for Statistical Computing). In terms of anti-VEGF-agent initiation, ranibizumab was preferred when initiating prior to 2014 and aflibercept thereafter. Both agents were used as per the Summaries of Product Characteristics of each drug in the first year and in the second year, with pro re nata as per clinician discretion. Switching to intravitreal dexamethasone was per treatment guidance provided by the National Institute for Health and Care Excellence Technology appraisal guidance (TA824).

### 2.3. Study Outcomes

The primary outcome was time from initial intravitreal dexamethasone injection to a positive visual outcome, taken to be an increase of at least 5 Early Treatment Diabetic Retinopathy Study (ETDRS) letters from baseline visual acuity (VA) recorded at 2 consecutive visits. Secondary outcomes included (i) time to increase of at least 10 ETDRS letters from baseline at 2 consecutive visits and (ii) duration at which positive visual response was sustained (defined as maintaining a VA gain of at least the defined thresholds—5 and 10 ETDRS letters—or subsequent treatment with intravitreal dexamethasone or anti-VEGF). Here, only patients who achieved the primary outcome were considered, and baseline was reset to the time point at which a positive visual response was achieved. All patients on the retinal intravitreal-therapy pathway had VA measured, as per standard ETDRS protocol. Increases in intraocular pressure (IOP) following treatment were evaluated, as well as whether patients were started on IOP-lowering medications or underwent a IOP-lowering procedure. We reported diabetic retinopathy standards at baseline, graded as per the English Screening Programme for Diabetic Retinopathy standards [19]. Briefly, retinopathies were graded into four levels: none (R0), background (R1; microaneurysms, retinal hemorrhages, venous loops, or any exudate in the presence of other non-referable features), pre-proliferative (R2; venous beading, reduplication, multiple-blot hemorrhages or intraretinal microvascular abnormality), and proliferative (R3) retinopathy. R3 was further classified into active proliferative disease (R3A; new vessels at the disc, elsewhere, pre-retinal, or vitreous hemorrhages, or pre-retinal fibrosis with or without tractional detachment) and stable treated proliferative disease (R3S). Maculopathy and photocoagulation were graded as absent (M0, P0) or present (M1, P1). M1 included the presence of exudates within one disc diameter of the center of the fovea, retinal thickening within one disc diameter of the center of the fovea, a group of exudates within the macula, or any microaneurysm or hemorrhage within one disc diameter of the center of the fovea only associated with a best VA of 20/40 Snellen or below. When gradings could not be assigned due to image quality, ungradable (U) was assigned.

### 2.4. Statistical Analysis

All data analyses were carried out with R (version 3.5.1) [20]. Hazards were modelled with Kaplan–Meier models [21]. Survival curves were plotted using the classical Kaplan–Meier estimator based on tabulation of the number at risk and number of events at each unique event time. To isolate efficacy of a single injection, data were censored at 6 months following initial injection and if subsequent intravitreal therapy was administered. All clinical data were recorded within an electronic medical-record application (OpenEyes Foundation), as previously described [22].

## 3. Results

### 3.1. Cohort Demographics and Clinical Features

Of 418 patients with DME who started intravitreal dexamethasone in the study period, 240 met the eligibility criteria for further analysis (Supplementary Figure S1). Patients included in the study were predominantly male (60.4%), with a median (range) age of 67 (29–90) years. More than half of all patients (53.4%) had >6 previous anti-VEGF injections recorded at baseline (Table 1). The mean (SD) baseline VA in this cohort was 56.0 (16.3) ETDRS letters, and 74.6% of patients had a baseline VA below 70 ETDRS letters, which is the legal lower limit for driving in the UK. Baseline retinopathy was variable, with similar incidences of each classification (R1 (36.3%), R2 (29.6%), and R3S (31.3%)). The majority of our cohort (70.4%) was pseudophakic.

### 3.2. Visual and Anatomical Outcomes Resulting from Initial Intravitreal Dexamethasone

In an initial analysis to assess the anatomical effects of the initial intravitreal dexamethasone injections over time, we analyzed monthly records of central subfoveal thickness for 6 months post-injection. This method of analysis, with a 10-day margin for each month, corresponds to the per-visit sampling approach that would have been adopted per protocol in the Phase 3 RCT for this treatment.

**Figure 1:**
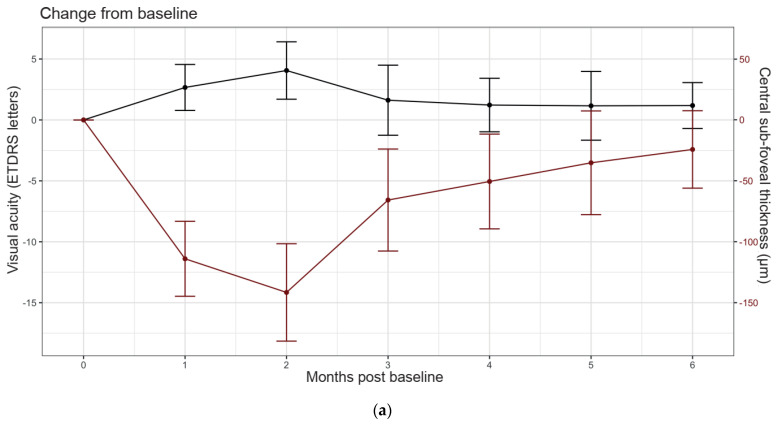

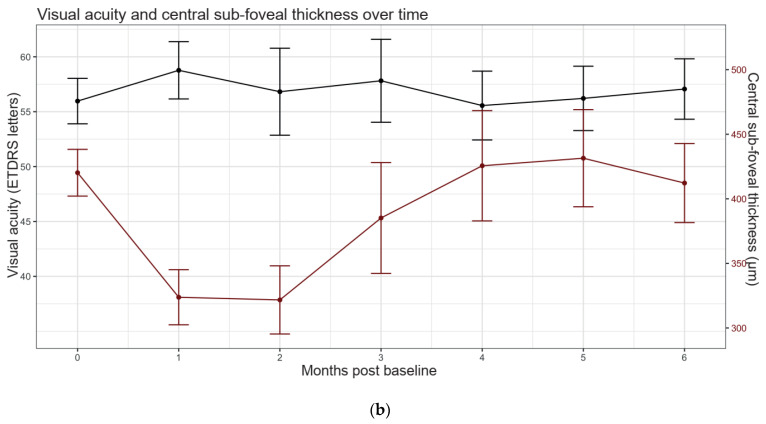
Monthly sampling of visual and anatomical outcomes up to 6 months following initial intravitreal dexamethasone. (**a**) Mean visual acuity (Early Treatment Diabetic Retinopathy Study (ETDRS) letters; black and central subfoveal thickness (μm; red) following initial intravitreal dexamethasone. (**b**) Mean change in VA and central subfoveal thickness from baseline. Error bars signify 95% confidence intervals.

**Figure 2:**
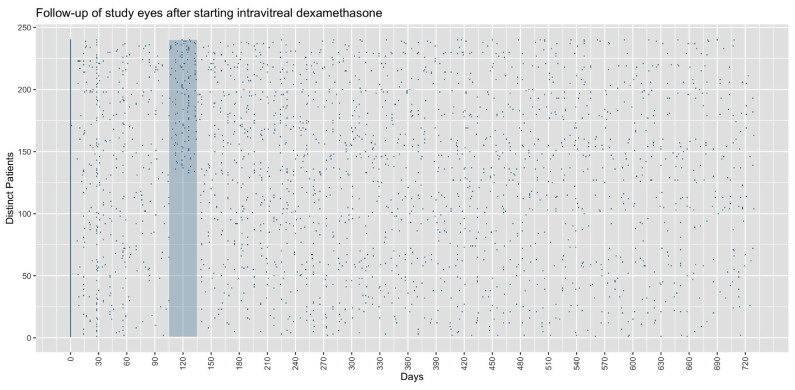
Data capture at monthly time points. Distinct clinical visits where visual acuity was measured are plotted (blue dots). *X*-axis depicts time following initial intravitreal dexamethasone and was restricted to the first 2 years. Blue-shaded area represents the 4-month time point—120 days post initial injection with a 10-day margin. Distinct patients represented along the *y*-axis and arranged so that patients with a clinical visit within the 4-month time point are at the top. Here it is demonstrated that an absent value does not suggest absence of follow-up. Of the 134 persons without a visual-acuity measurement at the 4-month time point, 100% (135/135) had a measurement following the 4-month period.

We found that mean central subfoveal thickness rapidly decreased within a month of treatment and remained decreased until month 2 (Figure 1a and Table 2). From month 3 onwards, mean central subfoveal thickness gradually returned towards baseline thickness, such that at month 5 and month 6 no significant change versus baseline was observed (Figure 1a,b). These early improvements in central subfoveal anatomy were not reflected as marked improvements in mean VA (Figure 1a and Table 2). We note a slight upward mean change from baseline in month 1 and month 2 (Figure 1b and Table 2). For the remaining months, mean VA remained stable post-injection and no mean change versus baseline was observed (Figure 1a and Figure 2b).

### 3.3. Interrogating Clinically Relevant Events following a Single Intravitreal Dexamethasone Injection with Time–Event Analysis

For the per-month sampling analysis, we allowed a 10-day time window each month following baseline to capture the scheduling of patient visits that may have been slightly offset to the monthly pattern of our analysis. Yet, we note that each month we captured only 29–60% of our baseline cohort for each time point in the VA analysis (Table 2). With 16–37% of patients captured each month, this effect was even more pronounced for the central subfoveal-thickness analysis. In an effort to better understand the missingness mechanism in our dataset, we plotted the time course of follow-ups in each study eye over the observation period (Figure 2). As expected for a real-world cohort wherein monthly examinations are not protocolized as with RCTs, there were considerable numbers of absent values at the monthly time points selected for our initial analysis of the data. At the month-4 time window (120 days, with a 10-day margin), out of 240 eyes 135 (56%) had no VA measurement. Of these, 135 (100%) had a VA measurement following the month-4 time point.

**Figure 3:**
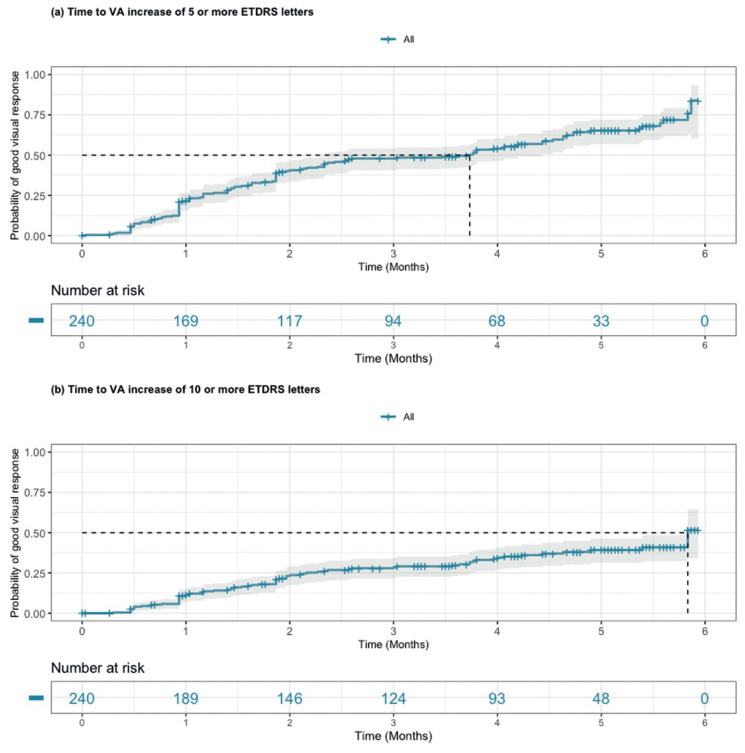
Probability of a positive visual outcome from an initial intravitreal dexamethasone injection. Kaplan–Meier modelling was carried out to estimate the probability of gaining (**a**) 5 or more ETDRS letters (Snellen equivalent 1 line) and (**b**) 10 or more ETDRS letters. Grey fill represents 95% confidence intervals. Tick marks indicate censored data, with remaining numbers at risk shown in the legend below.

An alternative to monthly sampling of data is time-to-event analysis, which can be used to estimate probabilities of clinically important events at given time points. We analyzed the time to a good visual response with two different thresholds, a VA of 5 and 10 ETDRS letters. To isolate efficacy of the first injection only, the follow-up period was limited to 6 months and any observations after subsequent intravitreal therapy were not considered. After a single dexamethasone injection, we found a chance greater than 75% for patients to achieve an improvement in VA of 5 ETDRS letters or more within 6 months (Figure 3a; median event time 3.63 months (95% CI 2.33–4.43)). Following initiation of intravitreal dexamethasone, 15 patients underwent cataract surgery. To account for potential confounding of visual outcomes, sensitivity analyses were carried out by estimating event probabilities without these 15 patients. Reassuringly, there was overlap in the resultant median event time (4.73 months (95% CI 3.8–7.23)). Setting a higher threshold of 10 or more letters, we found that there was a chance greater than 50% of achieving this positive clinical outcome within 6 months of the first dexamethasone injection (Figure 3b; median event time 5.83 months (95% CI 5.33–6.33)). Of the 64 patients that experienced an increase of 10 letters or more, 64% (41/64) had a VA below 70 ETDRS letters in both eyes at the initial intravitreal dexamethasone treatment, translating to a certifiable visual impairment in the UK. Following injection, in 34% (14/41) of these patients the VA increased to ≥70 ETDRS letters or above.

Out of 240 patients started on intravitreal dexamethasone, 130 patients achieved an improvement in VA of 5 ETDRS letters or more. When only considering the 130 patients who achieved this outcome, the probability of sustaining this response beyond 4 months after the event was 50% (Supplementary Figure S2a). Failure to sustain their positive visual response was observed for most of these patients (119/130). Similar results were seen for the 10-ETDRS-letter threshold, with a median duration of sustained response of 3.6 months (95% CI 3.03–5.13) (Supplementary Figure S2b), indicating that a stronger response did not result in longer duration.

Interestingly, for less than half (46% (55/119)) of those who failed to sustain a positive response was retreatment anticipated (with Eylea (n = 12), Lucentis (n = 1), or 32 Ozurdex (n = 32)) and administered before the VA benefit was lost. As patients with an initial response to intravitreal dexamethasone are eligible for retreatment, we wanted to understand the time to retreatment with intravitreal dexamethasone. The median time to retreatment in the cohort was 10.4 months (95% CI 8.5–13.3) (Supplementary Figure S3).

### 3.4. Intraocular Pressure Changes following Treatment

Following initiation of intravitreal dexamethasone, an IOP of 25 mmHg or more and 35 mmHg or more was observed in 7.9% (19/240) and 0.4% (1/240) of study eyes, respectively (Table 3). Herein, all patients with raised IOP following intravitreal dexamethasone received topical IOP-lowering therapy (7.9%, 19/240). One patient underwent an IOP-lowering procedure following injection; however, this patient had a prior diagnosis of open-angle glaucoma and underwent trabeculectomy without steroid-induced increases in IOP prior to initiation of intravitreal dexamethasone.

## 4. Discussion

Within 6 months after an initial intravitreal dexamethasone injection in a large UK tertiary eye-care center, most patients can be expected to have a positive visual outcome. A recent UK-based prospective cohort study reported improvement and maintenance of VA outcomes in treatment-naive DME patients started on aflibercept therapy, particularly so in the subgroup presenting with good VA at baseline. Importantly, more than 60% of treatment-naive patients in their cohort had a VA of ≥70 ETDRS letters (mean VA 71.4 letters), whereas in our cohort, in which patients were pretreated, more than 70% were below this threshold [23]. Zarranz-Ventura and colleagues investigated the differential response to intravitreal dexamethasone of treatment-naive and previously treated DME patients. Patients who were previously in their study had lower baseline VA compared to those in our study (37.5 letters) and showed less improvement [24]. Both study findings highlight the importance of early identification and treatment of DME patients.

As the implant biopolymer is designed to release dexamethasone into the vitreous humor over a finite period of time, it is unsurprising that we report a mean response duration of around 4 months for the positive visual outcomes observed. Earlier pharmacodynamic analyses of dexamethasone implants in monkeys showed that dexamethasone levels in the vitreous fell below the detection limit after 6 months [25]. This translated into biochemical effects in the retinal tissues for the same amount of time. Functional visual outcomes were not assessed in this study. In clinical trials, variable re-treatment intervals were chosen for dexamethasone implants, ranging from four to six months [9,26,27].

### 4.1. Time to Retreatment

Patients treated with intravitreal dexamethasone who have experienced an initial response and (in the clinician’s opinion) may benefit from retreatment without being exposed to significant side effects should be considered for retreatment. According to European treatment guidelines, retreatment may be performed after approximately 6 months if the patient experiences decreased vision and/or an increase in retinal thickness, secondary to recurrent or worsening DME. In a retrospective chart-review study of Canadian patients with macular edema, the mean (standard error) reinjection interval for the second dexamethasone implant in DME (n = 34 eyes) was 5.8 (0.5) months. Although this was the longest interval for all types of macular edema included in the study (DME, as well as retinal-vein occlusion (n = 30) and uveitis (n = 23)), the treatment pattern was much closer to the treatment pattern of ≥6-month intervals chosen in the Macular Edema Assessment of implantable Dexamethasone in diabetes (MEAD) study, a randomized sham-controlled Phase 3 trial program, than what was observed in our center [8].

Zarranz-Ventura and colleagues reported a mean retreatment injection interval of 10 months for pre-treated DME patients after an initial injection in their retrospective cohort [24]. In our cohort, less than 50% of patients went on to receive retreatment by 10.5 months. The median duration for an ETDRS-letter response ≥ 5 was 4 months. This may explain the observation that plans for retreatment in over half of the patients were only initiated after the VA benefit gained with dexamethasone treatment was lost, which is in line with the European guidelines. It remains to be determined whether the requirement for a decrease in vision before retreatment is initiated has any impact on patient outcomes, burden, and quality of life. However, earlier identification, treatment initiation, change of treatment in recalcitrant cases, and retreatment could help sustain the positive visual response observed for a considerable proportion of patients in our cohort that were certifiably visually impaired or below the legal limit to drive in the UK prior to intravitreal steroid treatment.

### 4.2. Survival Analysis as an Approach to Represent Real-World Data

Within clinical-trial frameworks or observational studies, various imputation models for missing data have been successfully applied to biostatistical-outcome analysis while retaining statistical power and avoiding bias [28]. However, patterns and mechanisms of missing data within health-record data do not permit techniques used in RCTs [29]. Patient health records may include missing data for intentional (e.g., the patient does not need follow-up) or unintentional (e.g., lack of routine checkup or follow-up) reasons. Therefore, missing data are common in routinely collected health data, and often missingness is informative [30].

We had a large data set of clinical DME data and therefore gave special consideration to the approach to missingness in our analysis. In real-world studies querying anti-VEGF efficacy in DME, missing data are reported to be 13%, 31%, 48%, and 65% after 1, 2, 3, and 4 years, respectively, and even up to 95% after 2 years in multicenter analysis [31]. However, this arises because routine clinical practice does not mandate assessment and treatment of study participants at pre-specified intervals (e.g., monthly visits), as is the case in RCT. This means that an absent data point within any time window post acquisition of the data does not necessarily equal missingness. However, the pattern of its absence cannot be assumed to be random, either. Consequently, if time-interval-based cohort sampling is applied to real-world data, this approach artificially increases missingness in the dataset for patients whose visits do not adhere to the required pattern.

As previously argued in the context of intravitreal-therapy efficacy in age-related macular degeneration [18], time–event analyses (such as Kaplan–Meier and Cox proportional-hazards regression) are an alternative to group means at arbitrary time points for approaching real-world clinical data with the advantage of (i) addressing some limitations of clinical-practice data as they make use of all available data, (ii) accounting for variable and biased follow-up duration, and (iii) evaluating clinically meaningful endpoints.

### 4.3. Intravitreal Dexamethasone in Phakic Patients

The National Institute for Health and Care Excellence Technology appraisal guidance (TA824) for dexamethasone intravitreal implant for treating diabetic macular edema, first published on 22 July 2015, specifically recommends treatment of pseudophakic DME with no provision for phakic patients. It is therefore notable that 29% (70/240) of our cohort was noted to be phakic at initiation of intravitreal dexamethasone. A portion of this sub-cohort is likely to represent patients that had cataract surgery planned to occur at the same time as or closely following the intravitreal dexamethasone injection. Indeed, 12 of 70 phakic patients underwent cataract surgery in the follow-up period. For the remainder, treatment of phakic eyes was likely to be informed by individual patient factors and emerging evidence for the efficacy of intravitreal-dexamethasone-phakic patients [15,32,33,34]. Indeed, an extension of the licensing of intravitreal dexamethasone to patients with DME has been proposed, and the data presented here would be in support of this trend.

### 4.4. Limitations

Limitations of observational studies of real-world clinical data are well recognized, encompassing various sources of variability of patient characteristics, treatment approaches, follow-up procedures, and outcome measurements. Although real-world studies lack experiment intervention and randomization to account for chance and confounding, as is featured in RCTs, they often benefit from larger sample sizes and greater heterogeneity, enabling a comprehensive understanding of therapeutic agents and a more accurate representation of the target population. As such, real-world studies are complements to clinical trials in generating the evidence base for treatment utilization, optimal patient management, and ensuring continued endorsement of therapeutics by regulators, payers, clinicians, and patients. Another limitation of this study is that known biomarkers of therapy response were not considered, including central macular thickness, subretinal fluid, subfoveal neuroretinal detachment, disorganization of retinal inner layers, hyperreflective foci, and photoreceptor integrity [35,36,37,38,39,40]. Certainly, one would expect these features to also be predictive of visual response in this cohort using these analyses. It should be noted that all VA measurements in this study were taken using standard ETDRS protocol, which can consistently be greater than the more widespread Snellen VA annotation by one or two lines [41]. It is also important to note that our dataset was unable to include 71 patients with insufficient follow-up. This is a potential limitation of this selection bias, wherein this sub-cohort does not represent a random sample of the target population.

## 5. Conclusions

This retrospective cohort study using electronic medical records of 240 UK patients with DME indicates that most patients can be expected to have a positive visual outcome following an initial injection of dexamethasone implants. Treatment effects subside with the degradation of the corticosteroid implant, and we observed a median response duration of around 4 months in our cohort. We use time-to-event analysis as an alternative method for the analysis of real-world data, preventing random exclusion of data and providing clinically relevant events. In current treatment guidelines, retreatment with intravitreal dexamethasone follows the loss of visual benefits with an improved understanding of when visual outcomes can be expected to last, which will hopefully be able to reduce lag to retreatment. For retrospective analyses of clinical-practice data, our findings demonstrate that time–event analysis can account for variable and biased follow-up duration and can be used to evaluate clinically meaningful categorical variables.

## Figures and Tables

**Table 1:** Demographic characteristics and clinical features of cohort. Baseline time point was taken to be time of initial intravitreal dexamethasone injection. Mean, median, minimum (Min), maximum (Max), standard deviation (SD), and interquartile range (IQR) are shown for (**a**) demographic and (**b**) clinical features (IDAOPI, Income Deprivation Affecting Older People Index; ETDRS, Early Treatment Diabetic Retinopathy Study; VA, visual acuity).

(a) Baseline Demography	
	*n* = 240
**Gender**	
Male	145 (60.4%)
Female	95 (39.6%)
**Age at recruitment**	
Mean (SD)	68.5 (9.88)
Median (IQR)	67 (13)
Min, Max	29, 90
**Ethnicity**	
Afro-Caribbean	48 (20.0%)
Caucasian	51 (21.3%)
Chinese	1 (0.4%)
Southeast Asian	90 (37.5%)
Mixed	5 (2.1%)
Unknown	45 (18.8%)
**Multiple deprivation index (decile)**	
1	6 (2.5%)
2	35 (14.6%)
3	53 (22.1%)
4	33 (13.8%)
5	38 (15.8%)
6	27 (11.3%)
7	14 (5.8%)
8	15 (6.3%)
9	9 (3.8%)
10	9 (3.8%)
Missing	1 (0.4%)
**IDAOPI**	
Mean (SD)	3.39 (2.33)
Median (IQR)	3.0 (3.0)
Min, Max	1.0, 10
Missing	1 (0.4%)
**IDACI**	
Mean (SD)	4.50 (2.26)
Median (IQR)	4.0 (3.0)
Min, Max	1.0, 10
Missing	1 (0.4%)
**(b) Baseline Clinical Features**	
	n = 240
**Baseline visual acuity (ETDRS letters)**	
Mean (SD)	56.0 (16.3)
Median (IQR)	58 (23)
Min, Max	0, 82
**Number of patients by ETDRS-letter category (%)**	
<70	179 (74.6%)
≥70	61 (25.4%)
**Baseline central foveal thickness (μm** **)**	
Mean (SD)	420 (142)
Median (IQR)	400 (170)
Min, Max	160, 950
**Appropriate follow-up of both CFT and VA**	
Complete follow-up	214 (89.2%)
Missing	26 (10.8%)
Lens status	
Phakic	70 (29.2%)
Pseudophakic	169 (70.4%)
Missing	1 (0.4%)
**Number of patients by baseline retinopathy grade (%)**	
R0	1 (0.4%)
R1	87 (36.3%)
R2	71 (29.6%)
R3A	5 (2.1%)
R3S	75 (31.3%)
U	1 (0.4%)
**Initial anti-VEGF agent (%)**	
Ranibizumab	96 (40.0%)
Aflibercept	71 (29.6%)
Bevacizumab	2 (0.01%)
Missing	71 (29.6%)
**Number of previous anti-VEGF injections**	
Mean (SD)	12.8 (9.07)
Median (IQR)	10 (13)
Missing (%)	69 (28.8%)
**Number of anti-VEGF injections by category (%)**	
<6	43 (17.9%)
6–10	45 (18.8%)
11–18	41 (17.1%)
>18	42 (17.5%)
Missing	69 (28.8%)
**Follow-up visits following baseline**	
Mean (SD)	15.5 (9.3)
Median (IQR)	13 (11)
Min, Max	4, 51

**Table 2:** Visual acuity and central subfoveal thickness at baseline and monthly up to 6 months in response to initial intravitreal dexamethasone. Mean, median, minimum (Min), maximum (Max), standard deviation (SD), and interquartile range (IQR) are shown for (**a**) visual acuity (Early Treatment Diabetic Retinopathy Study letters (ETDRS) letters) and (**b**) central subfoveal thickness (µm) at baseline and 6 months following baseline.

**(a)**							
	0	1	2	3	4	5	6
	(N = 240)	(N = 144)	(N = 90)	(N = 71)	(N = 107)	(N = 94)	(N = 136)
Absolute visual acuity (ETDRS letters)							
Mean (SD)	56.0 (16.3)	58.8 (15.8)	56.8 (18.9)	57.8 (16.0)	55.6 (16.4)	56.2 (14.3)	57.1 (16.2)
Median (IQR)	58 (23)	61 (19)	61 (24)	60 (20)	60 (23)	60 (20)	60 (24)
Change in visual acuity (ETDRS letters)							
Mean (SD)	0 (0)	2.67 (11.5)	4.06 (11.2)	1.62 (12.1)	1.22 (11.5)	1.16 (13.8)	1.18 (11.1)
Median (IQR)	0 (0)	2.0 (9.3)	4.0 (12)	1.0 (9.0)	2.0 (11)	1.0 (9.8)	0 (13)
**(b)**							
	0	1	2	3	4	5	6
	(N = 240)	(N = 90)	(N = 55)	(N = 39)	(N = 51)	(N = 54)	(N = 90)
Absolute central sub-foveal thickness (μm)							
Mean (SD)	420 (142)	324 (102)	322 (97.6)	385 (132)	426 (152)	432 (138)	412 (146)
Median (IQR)	400 (170)	310 (110)	310 (150)	390 (200)	410 (150)	420 (190)	390 (180)
Change in central sub-foveal thickness (μm)							
Mean (SD)	0 (0)	−114 (147)	−142 (148)	−65.7 (129)	−50.5 (138)	−35.2 (156)	−24.2 (152)
Median (IQR)	0 (0)	−72 (120)	−110 (170)	−48 (100)	−32 (180)	−24 (180)	−12 (140)

**Table 3:** Intraocular-pressure parameters in study eyes. Intraocular pressure (IOP) being raised at any visit during the study following initiation of intravitreal dexamethasone is shown, as well as the use of IOP-lowering medications and procedures following baseline.

	n (%)
IOP 25 mmHg or more during the study	19 (7.9%)
IOP 35 mmHg or more during the study	1 (0.4%)
IOP increase of 10 mmHg or more from baseline	0 (0%)
Use of topical IOP-lowering medication	19 (7.9%)
Procedure for IOP control	1 (0.004%)

## Data Availability

D.J.F. (Moorfields Eye Hospital NHS Foundation Trust and UCL Institute of Ophthalmology) has had full access to all the data in the study and takes responsibility for the integrity of the data and the accuracy of the data analysis. Fu conducted and is responsible for the data analysis.

## Supplementary Materials

The following supporting information can be downloaded at: https://www.mdpi.com/article/10.3390/jcm12123878/s1, Figure S1: Consort diagram; Figure S2: Durability of positive visual response after the initial dexamethasone injection; Figure S3: Time to intravitreal dexamethasone retreatment.
