# Supplementary material for: Visual and Anatomical Outcomes of a Single Intravitreal Dexamethasone in Diabetic Macular Edema: An 8 Year Real-World Study
Source: J Clin Med. 2023 Jun 6;12(12):3878. doi: 10.3390/jcm12123878 (PMC10299241; doi:10.3390/jcm12123878)
Supplement: Supplementary file 1 [file jcm-12-03878-s001.zip › Supplementary Figure S1.pdf]

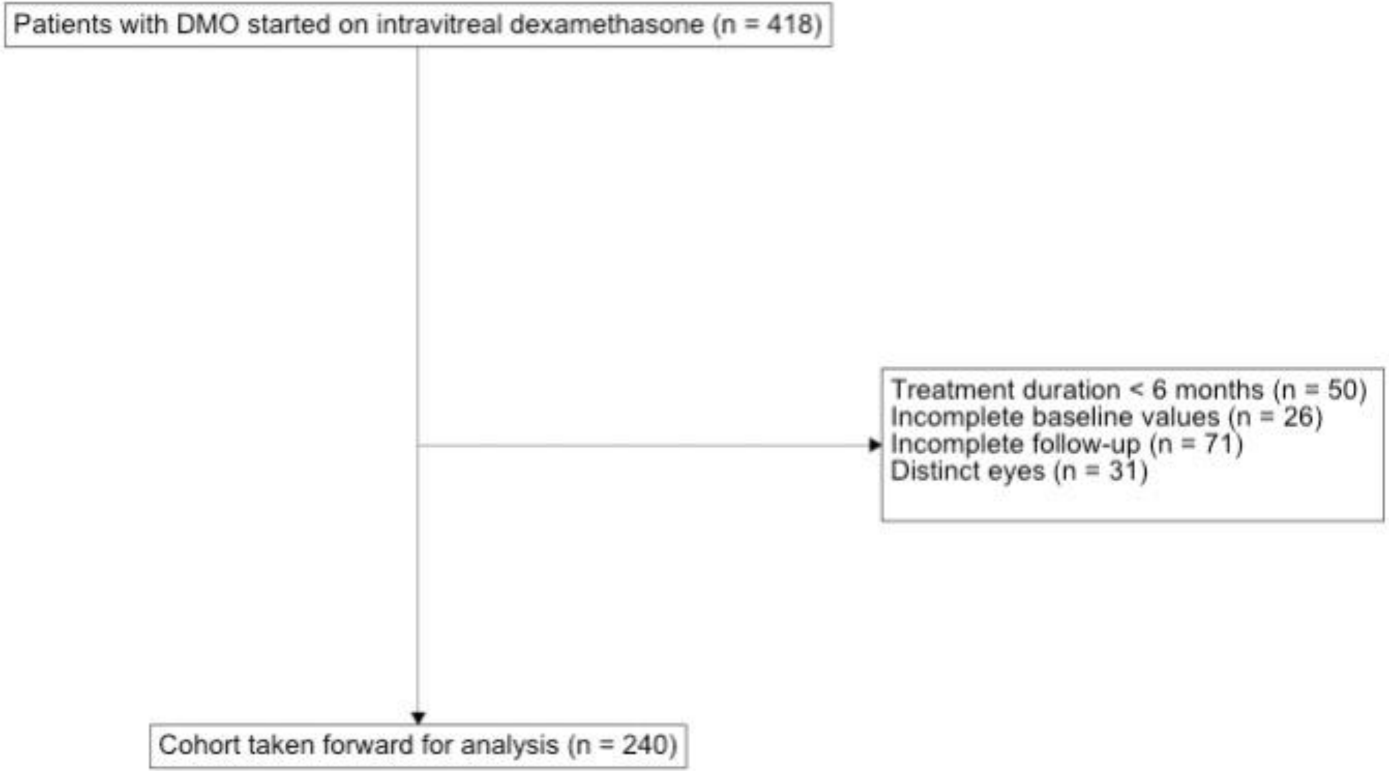
Patients with DMO started on intravitreal dexamethasone (n = 418)
Treatment duration < 6 months (n = 50)
Incomplete baseline values (n = 26)
Incomplete follow-up (n = 71)
Distinct eyes (n = 31)
Cohort taken forward for analysis (n = 240)
