# Supplementary material for: Visual and Anatomical Outcomes of a Single Intravitreal Dexamethasone in Diabetic Macular Edema: An 8 Year Real-World Study
Source: J Clin Med. 2023 Jun 6;12(12):3878. doi: 10.3390/jcm12123878 (PMC10299241; doi:10.3390/jcm12123878)

(a) Duration of positive visual response (benefit of 5 letters or more)

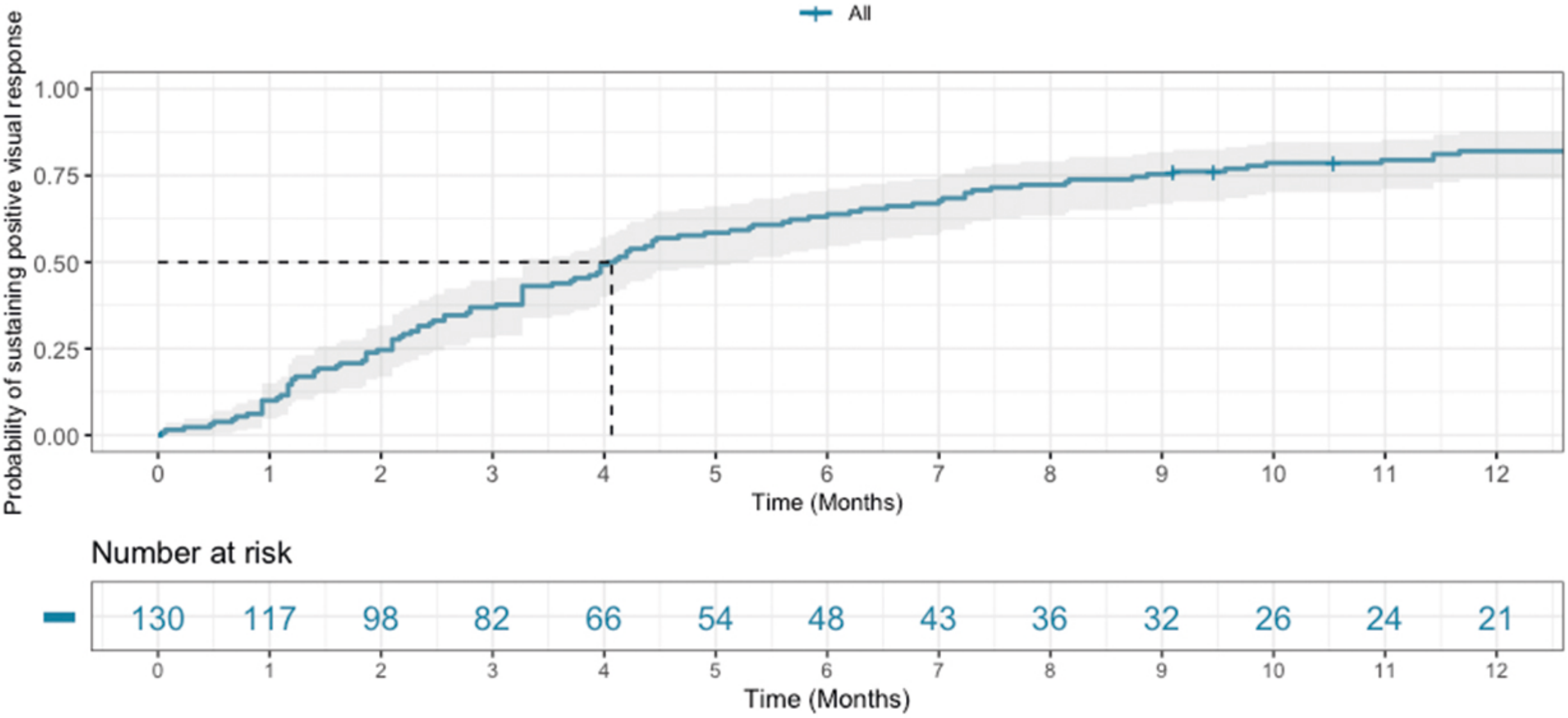

(b) Duration of positive visual response (benefit of 10 letters or more)

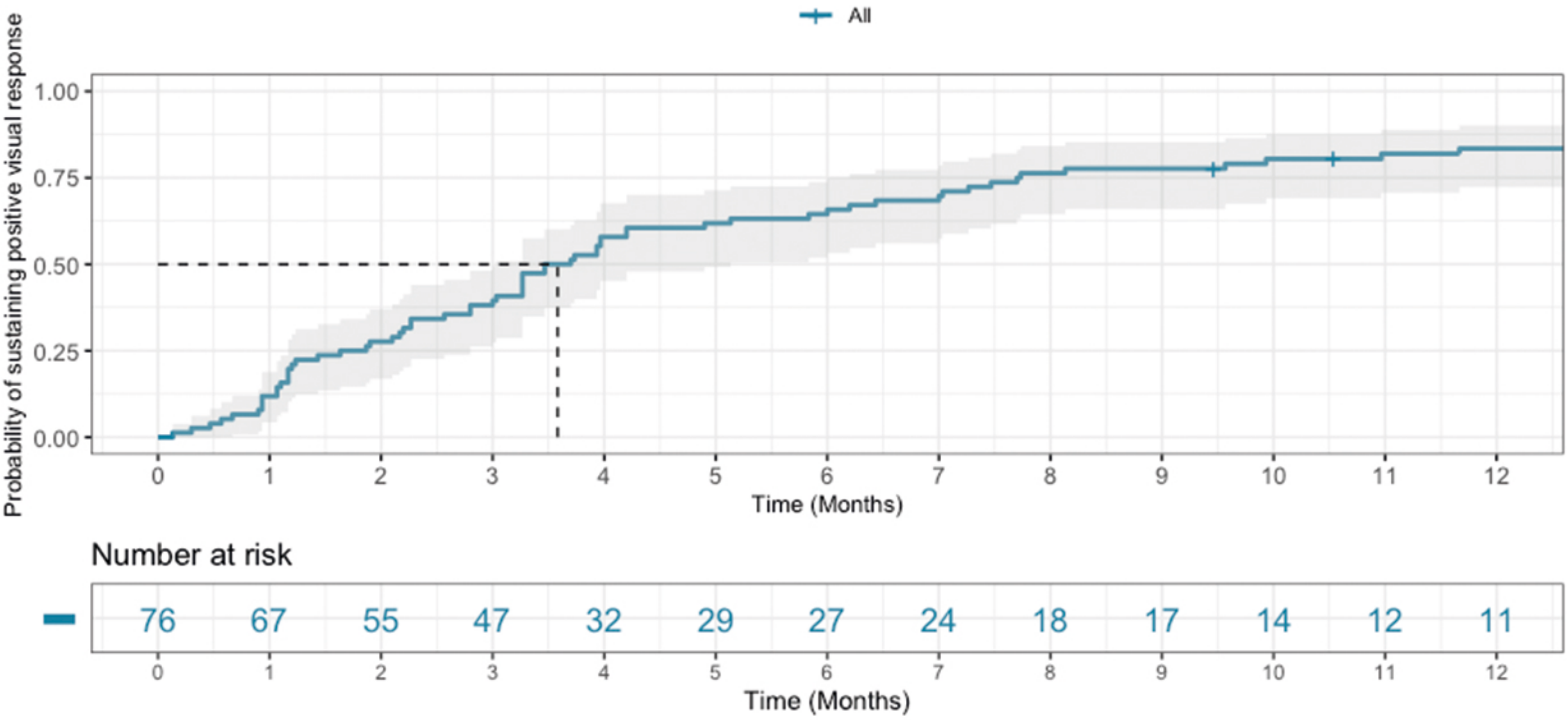

Supplement: Supplementary file 1 [file jcm-12-03878-s001.zip › Supplementary Figure S2.pdf]
