# Supplementary material for: Visual and Anatomical Outcomes of a Single Intravitreal Dexamethasone in Diabetic Macular Edema: An 8 Year Real-World Study
Source: J Clin Med. 2023 Jun 6;12(12):3878. doi: 10.3390/jcm12123878 (PMC10299241; doi:10.3390/jcm12123878)
Supplement: Supplementary file 1 [file jcm-12-03878-s001.zip › Supplementary Figure S3.pdf]

An Abbvie-MEH collaboration

Report 1: Real-world outcomes of a single intravitreal dexamethasone in DMO

Livia Fäs, Amit Mishra, Veronika Lipkova, Robin Hamilton, Sobha Sivaprasad, Dun Jack Fu

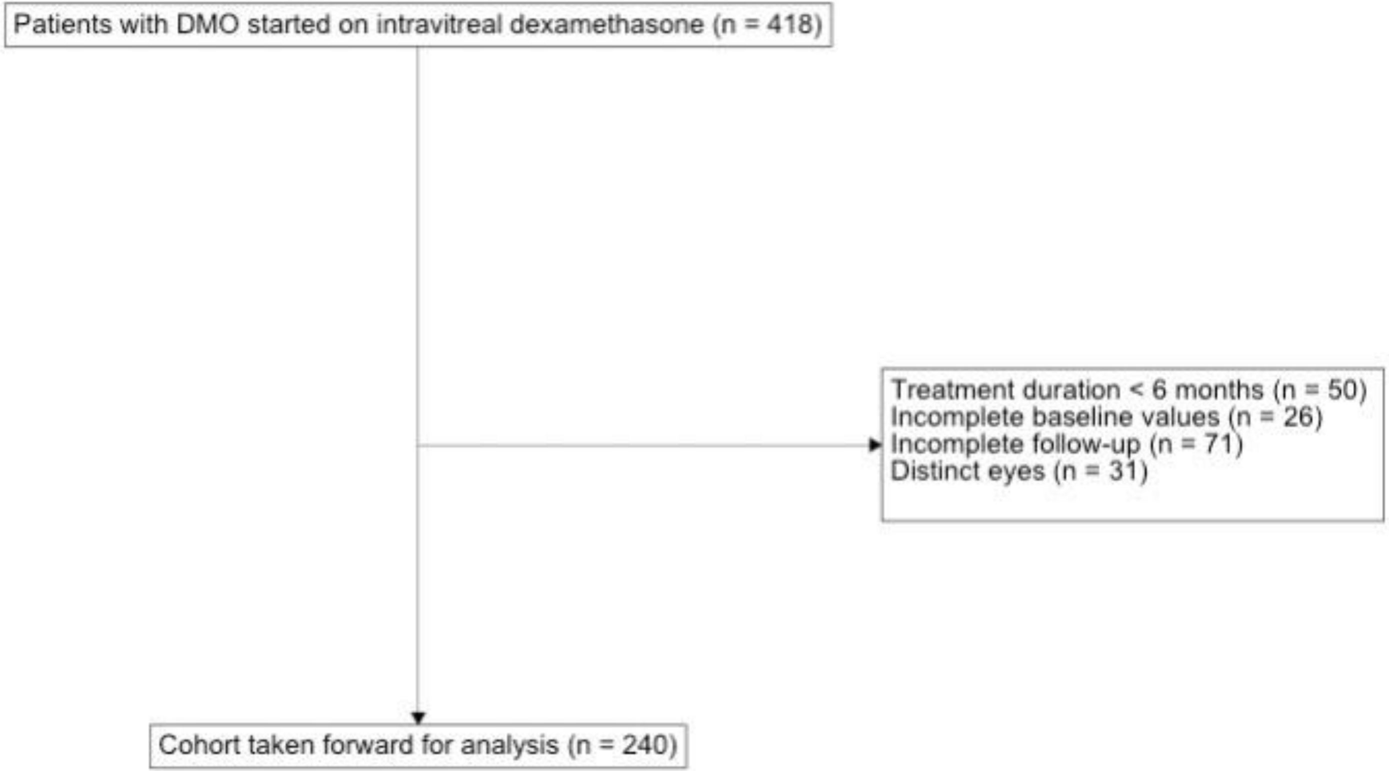
Patients with DMO started on intravitreal dexamethasone (n = 418)
Treatment duration < 6 months (n = 50)
Incomplete baseline values (n = 26)
Incomplete follow-up (n = 71)
Distinct eyes (n = 31)
Cohort taken forward for analysis (n = 240)

(a)	Overall (N=240)
**Gender**	
Male	145 (60.4%)
Female	95 (39.6%)
**Age at recruitment**	
Mean (SD)	68.5 (9.88)
Median [Min, Max]	67.0 [29.0, 90.0]
**Ethnicity**	
Afro Caribbean	48 (20.0%)
Caucasian	51 (21.3%)
Chinese	1 (0.4%)
Mixed	90 (37.5%)
South East Asian	5 (2.1%)
Unknown	45 (18.8%)
**Multiple deprivation index (decile)**	
1	6 (2.5%)
2	35 (14.6%)
3	Overall (N=240)
4	
5	145 (60.4%)
6	95 (39.6%)
7	
8	68.5 (9.88)
9	67.0 [29.0, 90.0]
10	
Missing	48 (20.0%)
**IDAOPI**	
Mean (SD)	3.39 (2.33)
Median [Min, Max]	3.00 [1.00, 10.0]
Missing	1 (0.4%)

(b)	Overall (N=240)
**Baseline Visual acuity (ETDRS letters)**	
Mean (SD)	56.0 (16.3)
Median [Min, Max]	58.0 [0, 82.0]
**Baseline VA greater than or equal to 70 ETDRS letters**	
<70	179 (74.6%)
70 or over	61 (25.4%)
**Baseline Central subfoveal thickness (microns)**	
Mean (SD)	420 (142)
Median [Min, Max]	400 [160, 953]
**Lens status**	
phakic	70 (29.2%)
pseudophakic	169 (70.4%)
**Baseline retinopathy**	
R0	1 (0.4%)
R1	87 (36.3%)
R2	71 (29.6%)
R3A	5 (2.1%)
R3S	75 (31.3%)
U	1 (0.4%)
**Number of anti-VEGF injections prior**	
Mean (SD)	12.8 (9.07)
Median [Min, Max]	10.0 [0, 50.0]
Missing	69 (28.8%)
**Number of anti-VEGF injections by category**	
< 6	43 (17.9%)
> 18	42 (17.5%)
11-18	41 (17.1%)
6-10	45 (18.8%)
Missing	69 (28.8%)

Visual & anatomical outcomes resulting from initial intravitreal dexamethasone

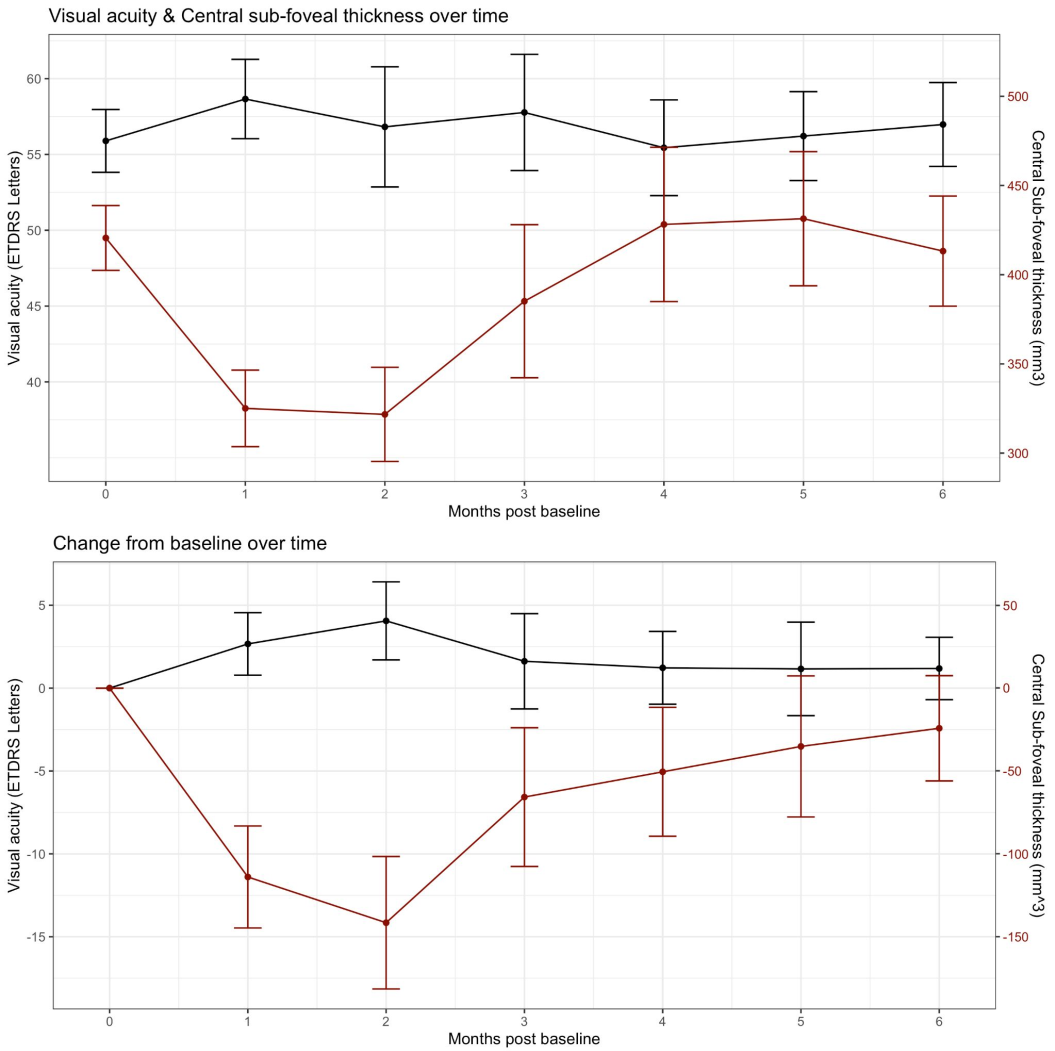
Visual acuity & Central sub-foveal thickness over time
Visual acuity (ETDRS Letters)
Central Sub-foveal thickness (mm3)
60
55
50
45
40
500
450
400
350
300
0 1 2 3 4 5 6
Months post baseline
Change from baseline over time
Visual acuity (ETDRS Letters)
Central Sub-foveal thickness (mm^3)
5
0
-5
-10
-15
50
0
-50
-100
-150
0 1 2 3 4 5 6
Months post baseline

(a)	0 (N=240)	1 (N=143)	2 (N=90)	3 (N=70)	4 (N=106)	5 (N=94)	6 (N=135)
**Absolute visual acuity (ETDRS letters)**							
Mean (SD)	55.9 (16.3)	58.7 (15.8)	56.8 (18.9)	57.8 (16.1)	55.4 (16.4)	56.2 (14.3)	57.0 (16.3)
Median [Min, Max]	58.0 [0, 82.0]	61.0 [0, 85.0]	61.0 [2.00, 85.0]	60.0 [2.00, 85.0]	60.0 [0, 78.0]	59.5 [5.00, 85.0]	60.0 [0, 85.0]
**Change in visual acuity**							
Mean (SD)	0 (0)	2.67 (11.5)	4.06 (11.2)	1.81 (12.1)	1.28 (11.5)	1.16 (13.8)	1.22 (11.1)
Median [Min, Max]	0 [0, 0]	2.00 [-40.0, 73.0]	4.00 [-33.0, 28.0]	1.50 [-33.0, 38.0]	2.00 [-52.0, 58.0]	1.00 [-50.0, 53.0]	0 [-35.0, 30.0]

(b)	0 (N=240)	1 (N=89)	2 (N=55)	3 (N=39)	4 (N=50)	5 (N=54)	6 (N=89)
**Absolute central subfoveal thickness (mm3)**							
Mean (SD)	421 (142)	325 (102)	322 (97.6)	385 (132)	428 (152)	432 (138)	413 (147)
Median [Min, Max]	400 [160, 953]	312 [150, 640]	313 [152, 528]	386 [165, 741]	412 [172, 820]	422 [210, 800]	388 [150, 839]
**Change in central subfoveal thickness from baseline**							
Mean (SD)	0 (0)	-114 (148)	-142 (148)	-65.7 (129)	-51.1 (140)	-35.2 (156)	-24.5 (153)
Median [Min, Max]	0 [0, 0]	-71.0 [-641, 190]	-107 [-801, 50.0]	-47.6 [-383, 219]	-32.3 [-403, 290]	-24.0 [-574, 358]	-13.0 [-651, 376]

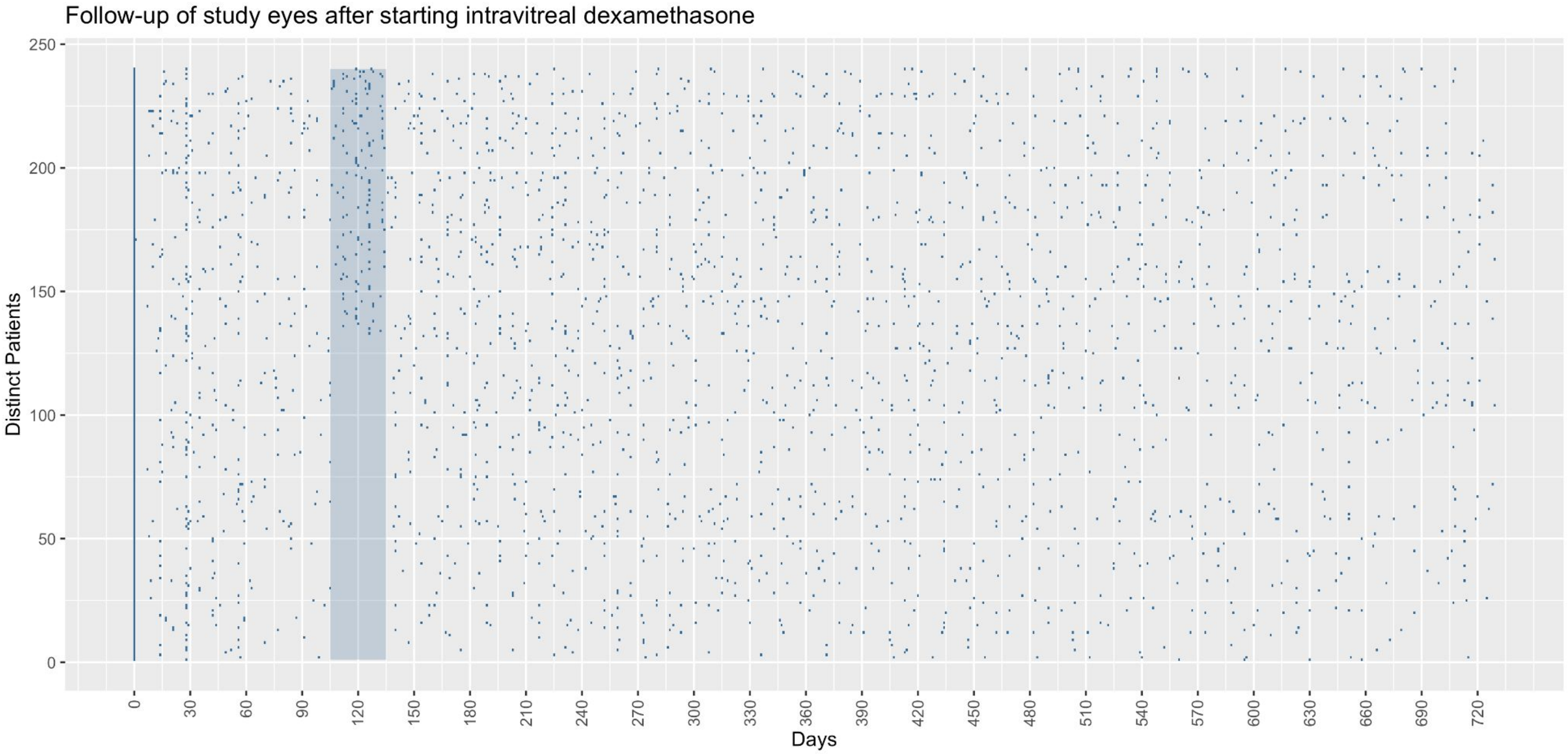
Follow-up of study eyes after starting intravitreal dexamethasone
Distinct Patients
Days
0
50
100
150
200
250
0
30
60
90
120
150
180
210
240
270
300
330
360
390
420
450
480
510
540
570
600
630
660
690
720

Using time-event analyses to interrogate visual and anatomical outcomes from a single intravitreal dexamethasone injection

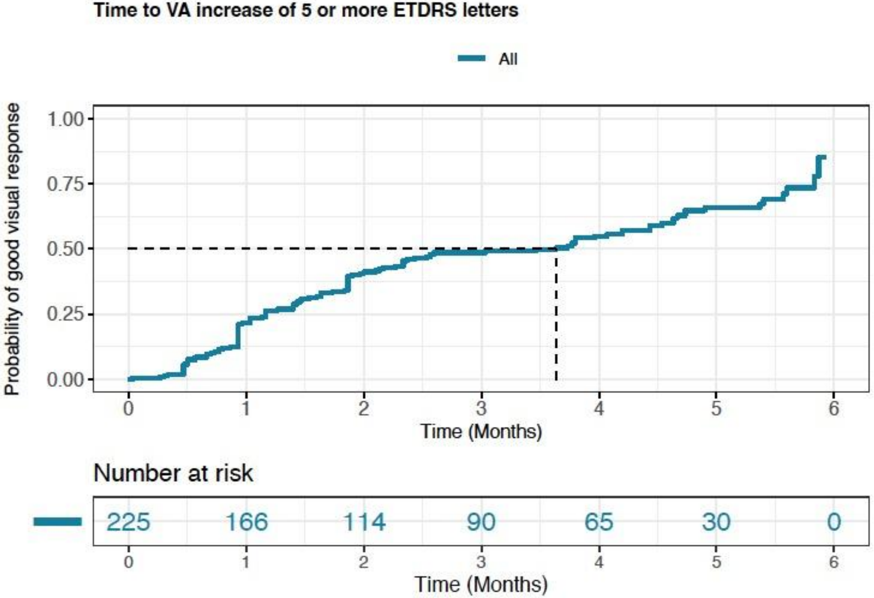

n	events	median	0.95LCL	0.95UCL
225	130	3.63	2.33	4.43

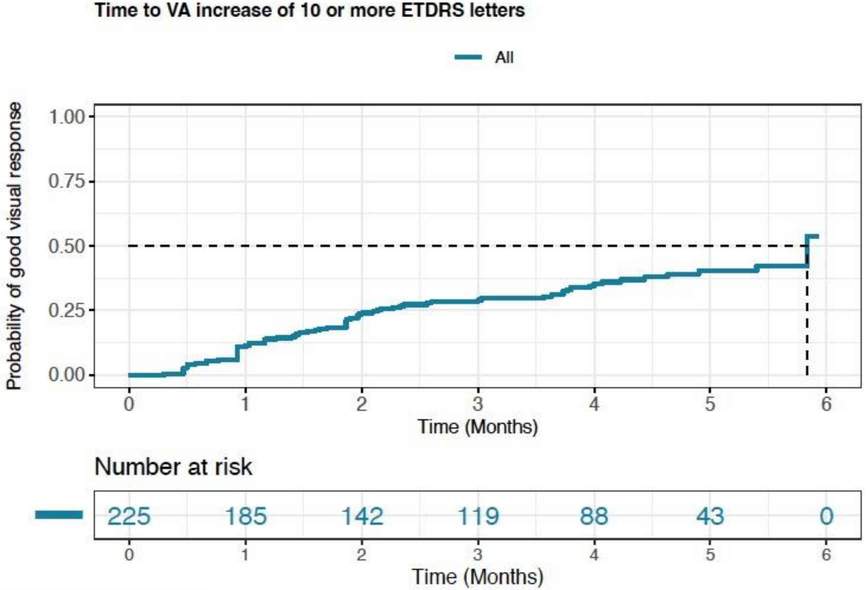

n	events	median	0.95LCL	0.95UCL
225	76	5.83	5.4	NA

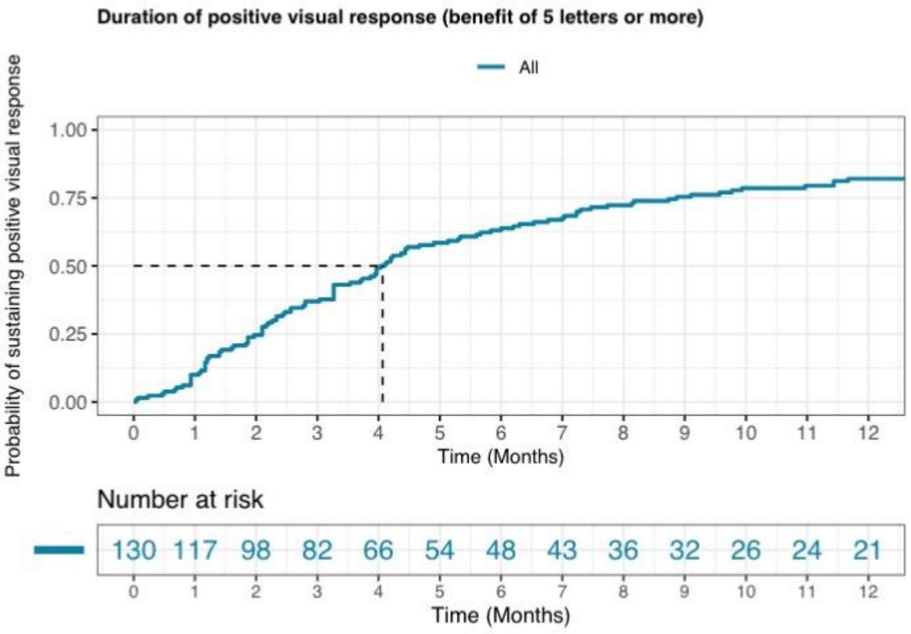

n	events	median	0.95LCL	0.95UCL
130	119	4.07	3.27	5.3

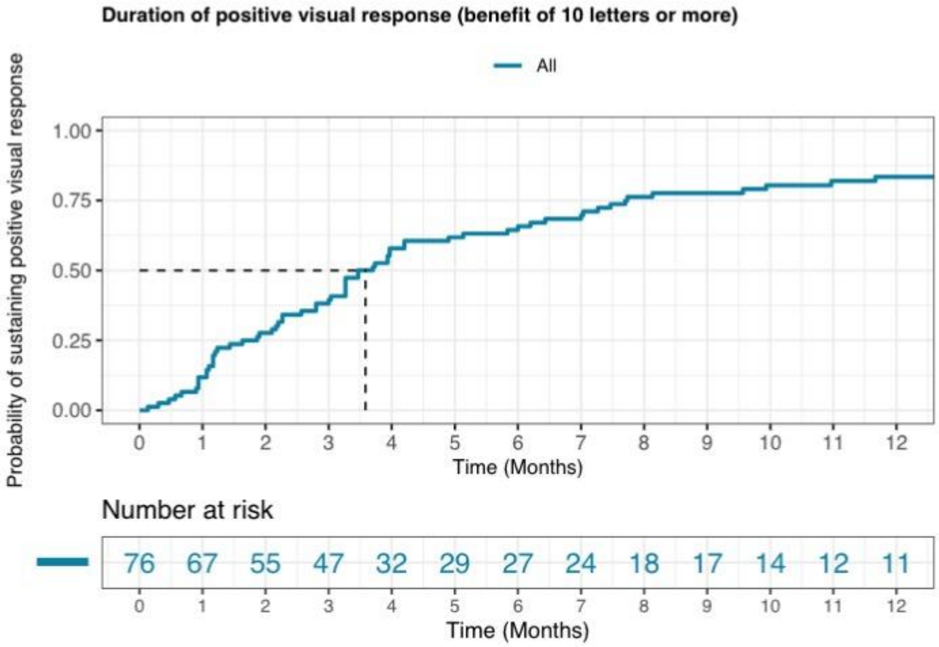

n	events	median	0.95LCL	0.95UCL
76	68	3.58	3.03	5.13

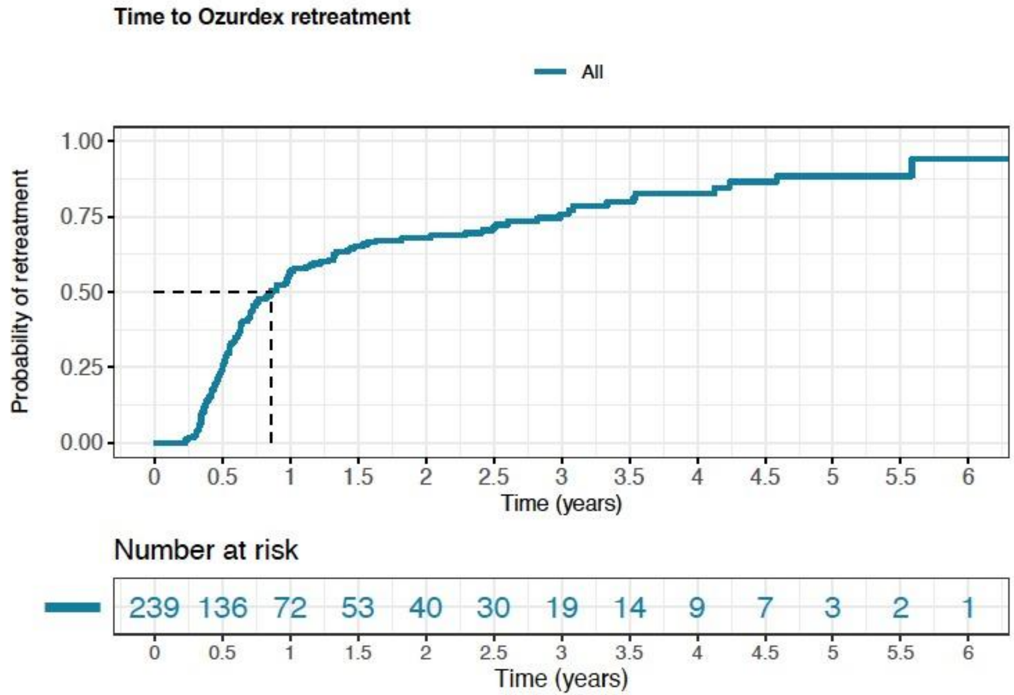

n	events	median	0.95LCL	0.95UCL
239	136	0.863	0.71	1.11
